# The Present Oxyphosphate Cement Situation

**Published:** 1912-01-15

**Authors:** W. V-B. Ames

**Affiliations:** Chicago


### THE PRESENT OXYPHOSPHATE CEMENT SITUATION.

BY W. V-B. AMES, D.D.S., CHICAGO.

I was asked, in effect, to present a paper as practical as I could make it on the uses of oxyphosphate cements, tinctured with only as much of the chemistry and manufacture as would tend to elucidate the subject. The most practical treatment of any subject embodying the use of manufactured materials must deal with those materials identified by their real names. Although in my position comparisons are distasteful, I cannot do other than use real names in mentioning materials in this effort to clear the atmosphere. Some investigations of these cements have, to the minds of most listeners and readers, lacked real value, because the identity of the materials has not been disclosed.

Happening to be the one individual *easily available*, in possession of a considerable knowledge of the manufacture of oxyphosphate ingredients, and possessed at the same time of the advantage of a fairly ripe experience in clinical dentistry, I trust that I will not appear too egotistical if I criticise more or less freely, and expose as fallacious much alleged information offered on this subject.

Information obtainable from dental chemistries, dental formularies, and magazine articles, generally seems to be based either on tradition or insufficient investigation. I prefer to comment but little on the information advanced by the manufacturing fraternity, although I will need to make indirect reference to several fallacies thus advanced, which will probably be recognized.

The oxids of zinc, calcium, iron, cobalt, manganese, and of some other metals not important to the subject, exhibit the property of combining with acids and acid-salts to form basic salts, when the oxid is presented to the acid in excess.

These basic salts have the property of acting as agglomerating media to hold together crystals or granules of the same oxid or other materials, the result being a more or less dense mass which may answer as a dental cement.

The crystalline forms of zinc oxid when reduced to an impalpable state furnished the best examples for cement making purposes.

Calcium oxid in any form acts violently with phosphoric and some other acids to form basic salts. It constitutes the chief active principle of the powders of the silicious oxyphosphates.

Copper oxids, black cupric and red cuprous, are valuable as cement makers when presented to an acid phosphate. The black cupric oxid proves more valuable than the cuprous.

Various forms of iron oxid manifest the cement making property, but are mainly useful in small percentages as modifiers. Brown oxid of manganese is quite similar to iron in its behavior and is only useful in the same small proportions.

Cobalt oxid in a pure state and of a proper oxidation is a cement ingredient of great value. Cobalt properly oxidized will react so violently with an acid phosphate solution that heat too intense to be tolerated will be developed, and *by* that definite setting a material is produced which is incomparably superior in density and strength to any cement mass I have yet observed.

While a cement produced by combining an acid phosphate solution and black oxid of copper is much superior n density and strength to the best possible oxyphosphate of zinc, so that therefore an oxyphosphate of zinc, may be improved as to texture according to the ratio in which copper oxide is properly added, any of these combinations may be vastly improved by the proper addition of a percentage of cobalt oxid. There has been much twaddle about danger resulting from arsenic carried by crude copper and cobalt, but it is merely twaddle. Cobalt oxid and cobalt salts of commerce are prepared mainly for use in the production of blue glazes and for a good blue glaze the cobalt must be freed of arsenic and other impurities. In the processes of refining, the removal of iron, lead, bismuth and nickel presupposes the removal of the last trace of arsenic so that if no other precautions were taken, this is sufficient assurance that arsenic free cobalt is obtainable. Copper being prepared electrolytically, purity here is easily secured.

The fact that the usual form of cobalt oxid has no cement making property and that the production of a cement-forming grade of oxid is apt to be the result of inspiration in addition to deliberation, easily accounts for this oxid being so little employed as an ingredient.

It may be said in this connection, in view of the infinitesimal amount of arsenic needed for pulp devitalization, that, were an ingredient dangerous from a standpoint of carrying arsenic, 30 % or even 3% it would be sufficient to preclude its use.

Of the principle oxids used in oxyphosphate cements, zinc oxid might be most easily expected to carry a trace of arsenic, as in the distillation of zinc and the sublimation of oxid, some arsenic might condense in either process. The producers of commercial zinc and zinc oxid need to, and do, look carefully to the elimination of arsenic which being much more volatile than zinc, is easily disposed of. It is then up to the cement maker to know of what he is using.

Pigmenting of zinc oxid may be harmful or beneficial, depending on the means employed. The necessary metallic oxids may be combined at a calcining heat with a resulting benefit. Any statement to the effect that without pigmenting oxids heat alone will give all desired shades from the natural creamy white to dark grays and browns, is simply ridiculous and the giving out of such statements by people who know better is beyond understanding.

In speaking of phosphoric acid as a cement ingredient it is always an acid phosphate solution which is really meant, as straight phosphoric acid is seldom used for oxyphosphate cement purposes. By the modification of phosphoric acid by phosphates, practically any working quality desired may be obtained. The early cement liquids were usually solutions of glacial phosphoric acid.

This glacial acid is the result of dissolving an indefinite amount of sodium in some form—depending on the manufacturer—in normal phosphoric acid. Cooking this solution down through the stage of being pyrophosphate solution to the condition in which it is a metaphosphate it becomes an indefinite mixture of metaphosphoric acid and sodium metaphosphate. The sodium is introduced to give a glassy stick, less deliquescent than would be a stick or lump of plain metaphosphoric acid. It is a coagulator of albumin, and therefore for that reason the solution as metaphosphate is not adapted to be used as a cement liquid. A solution of it can, however, be converted back into normal phosphoric acid and normal sodium phosphate by boiling with an excess of water, and this solution is not a coagulator of albumin, and may be used as a cement liquid when reduced to a proper specific gravity.

This explanation of the nature of glacial phosphoric acid and liquids resulting from boiling its solution in water seems advisable, as many early attempts at cement making were based on its use, and as many formulas have been published in which this indefinite commercial glacial acid is specified. If a plain sodium phosphate solution is of sufficient value for the purpose to call for its preparation, the proper method is to add a proportion of sodium carbonate or phosphate ascertained to be the proper amount, to a definite weight of orthophosphoric acid of a definite specific gravity, instead of buying an indefinite glacial phosphoric acid which will vary as purchased from different manufacturers, and probably at different times from the same manufacturer.

I dilate to this extent, not because it is highly useful as a cement ingredient for oral purposes, but because it is a useful solution for a cement for technical purposes as making of models, and other masses not to be subjected to moisture. A cement produced by mixing this solution with a good grade of heavy zinc oxide will have fair strength in the dry state, but will, at best, be a porous mass and as the agglomerating basic sodium phosphate is slowly soluble in water, such a mass would be of little use under oral conditions.

The rather prevalent belief that an oxyphosphate cement filling is not dependable at the cervical margin is largely the result of observation of approximal fillings made from cements compounded with an acid solution of sodium phosphate. These failures were not from solution or breaking down of the cement at this point, but from a leak created before the operation was dismissed as finished. Because of the lack of crispness during the setting there is the danger of drawing the cement away from the cervical margin in finishing or by the removal of the dam without cutting the septum occuping the space. It has often been ventured that such failures too often are the result of slovenly cavity preparation and moisture at the cervical region.

While sodium phosphate entire is not a desirable modifier of phosphoric acid for oxyphosphate cement for oral purposes, this phosphate does impart desirable working qualities and has also a salutary control of shrinkage and expansion when added in small percentage along with non-alkaline phosphates, such as aluminum, zinc, magnesium, and some others. These none-alkaline phosphates are depended on by the makers of the cements which have been found reliable. This reliability comes from density and imperviousness and these properties are attributable to the comparative integrity and insolubility of the basic phosphate formed in cement masses.

Sodium and other alkaline phosphates in cement give the possibility of a hastened setting by application of heat, which fact contraindicates the use of some descriptions of liquid in mid-summer and renders them advisable for winter use. There have recently appeared some tirades against sodium phosphate as a cement ingredient in one of which the hygroscopic property of sodium chlorid is cited as an argument against the use of sodium phosphate. More sense in placing a ban on calomel because corrosive sublimate is a powerful poison. Some alleged analyses have appeared in the same connection which scarcely deserve notice, but which may disturb the understanding of some, in which it is claimed that Justi's liquid contains 68%, Harvard 65, Caulk's 59, Ames 55 and Fellowship 50 of sodium phosphate.

As analyses these figures are ridiculously faulty, or as guesses they are ridiciously wide of the mark. In none of these liquids is there any such proportion. With some no more sodium phosphate would be found than a careless analyst would obtain from unclean glass ware.

On variously combining phosphates with phosphoric acid and water depends the differences of setting qualities, shrinkage and expansion, and as has been stated, the comparative integrity of mass. Imperviousness (non-porosity) is practically another manner of speech for integrity of mass. Porosity may be present —it might be said—in any moderate degree and not absolutely banish a cement from the usable category, while beyond a very circumscribed limit, shrinkage or expansion does place a cement outside of the usable category.

In other words, slight porosity in a cement as indicated by a vague pink tint after submission to eosin stain, is not a fatal fault, while an easily perceived shrinkage as indicated by eosin solution passing between a cement mass and the wall of a glass tube, or as shown in a Wedelstedt tube by measurements made by a Black micrometer, ought, I believe, to be considered as inviting failure. Cements which expand seriously may (and are apt to) show porosity because of a faulty crystallization. Expansion easily discernable in a cement may not, for some purposes, be fatal, yet an easily discernable expansion cannot be said to be a "positive advantage" in any use whatever. In a paper some years since, I endeavored to make it plain that expansion is due to the taking up of water because of a deficiency of water in the modified acid for proper crystallization. It was stated that an excess of water above the amount needed for crystallization would result in the giving off of water and a resulting shrinkage, and that the taking up of water for proper crystallization would result in expansion. Thus shrinkage and expansion may be regulated in compounding a cement liquid by adjusting the proportion of water, having in mind that for most purposes about a certain manipulation and consistency of mix will be recognized as proper. A too thin mix from a given formula will show expansion, whereas a very stiff mix will show slight shrinkage, the very thin mix and very stiff mix both being illadvised. The evident reason for these two observed results is that in each case the formation of the different grades of basic phosphate require different proportions of water for crystallization.

By easily made tests, with cements which I will mention in the order of their introduction, results such as I describe are obtained. In all tests the conditions under which the cement must exist in use in the mouth ought to be adopted in carrying on tests out of the mouth.

I do not feel that actual formulas need concern us in this connection, my understanding being that you are desirous of being told the result of my observations of the cements in most general use.

Justi's cement has held a place in dental procedures for more than 30 years, and must, therefore, be possessed of unusual merit. The claim, however, that the formula of this cement has not been changed in 37 years, I do not consider as entirely commendable. In fact the absence of crystals in the liquid as furnished now for some years, indicates a change, as the early product usually contained precipitates. Its producer was one of the earliest to depart from the sodium phosphate formula. This was the first oxyphosphate of zinc cement, unless it was the Rostaing, setting with the proper crispness to insure cervical margin safety. Eisfelder's, another German product, is very similar to the Justi and has been on the market for about the same period.

These cements, kept dry for a sufficient time for setting before being subjected to moisture, give a mass which is very slightly porous, show exceedingly well in adhesion tests, and show a barely perceptible expansion. These desirable qualities, expansion practically nil and exceedingly good adhesiveness, are dependent on the liquid element, the powder being rather coarse heavy zinc oxid.

Harvard cement, now in use nearly 20 years, is even slower of setting than the Justi, and requires a longer protection from moisture. This slower setting is partly from modification of acid and partly from modifications of the zinc oxid. Phosphates and other oxids are incorporated in the powder for good reason, we will grant, one result being a retarded setting. Harvard Quick Setting Inlay Cement is very little quicker than Harvard regular. The liquid is labeled pyrometaphosphatische dentinogen. Since a pyrometaphosphoric compound would be somewhat caustic and a serious irritant, I do not believe that the material is as bad as its label would indicate. This cement, protected from moisture for a half hour or more gives a mass which will show only slight porosity after subjection to eosin. A considerable expansion is inevitable with this cement after a stiff mix properly subjected to moisture and a greater expansion after a crown setting mix. Dr. Kulka, in "Items of Interest," May of last year, says "all cements show on their exposed surface a tendency to protrude in a rounded shape as soon as they are placed in the cavity." This statement and others in his article would indicate that he is familiar with no other cement than the Harvard, unless he uses Lynton, the sale of which was attempted on this side some years since. This cement expands more than Harvard. Adhesion of Harvard cement is quite satisfactory, as evidenced by tests and the testimony of users. The marked expansion is attributable mostly to powder modification. Careful dilution of the liquid with distilled water has resulted in a more satisfactory combination in the hands of a few operators. A more pratical modification for the cure of the marked expansion would be the elimination of phosphates from the powder by the manufacturer.

Silex Email, a French product, introduced during the latter years of the last century, would seem to be intended by the makers principally for the filling of cavities, as the powder is very coarse. For filling purposes it has proved dependable.

The next calling for chronological mention are the Ames cements of the oxyphosphate of zinc class. When somewhat more than twenty years since the writer became impressed with the practicability of the cemented metallic inlay, there arose the desirability of a different cement than was then obtainable. The Justi oxyphosphate of zinc happened to be the one relied on for crown setting and for all other purposes requiring attachments for long periods of time. Crowns set with that material thirty years ago are in acceptable condition to-day, as far as the cement attachment is concerned, and gold inlays of the burnished matrix type twenty years in service bear evidence of the reliability of this cement. However, when we became impressed with the practicability of the cemented metallic inlay and the porcelain inlay as made twenty years since under selected conditions, there arose a seeming need of a cement combining a finer and smoother powder, a somewhat quicker setting, and, if practicable, a cement with what we are in the habit of calling "hydraulic" properties. Experimentation on our part developed the Ames Special crown and Bridge and Ames Special Inlay Cements. Since quick setting, and the property of setting better when subjected to moisture during the setting process appealed to me and many others, and seemed clinically practicable, these cements have been mostly used in the combination of "C" liquid with variously compounded and tinted powders.

These cements as compounded with the "C" liquid do give prompt setting and the surplus, which should be left at any joint until setting has progressed to a leathery stage, may to advantage then be subjected to moisture, i. e., the saliva may be allowed to come in contact with this surplus, which in a few minutes will be sufficiently crisp to be removed in a few sections in a cleanly manner. The cement within the joint will then be far past being damaged by moisture, and should have moisture for complete and proper setting.

The makers of the so-termed hydraulic cements have been often misrepresented as to their claims and advices in regard to this property in cements. Dr. Prinz, in his Dental Formulary, gives an erroneous impression in a foot note in which he says: "Hydraulic cements give better results, according to Ames, if the cavity is slightly moistened with water after it has been dehydrated with alcohol."

I have never advised placing cement upon a surface actually wet, as that would represent much more moisture than would be taken up by an ordinary mass of cement during crystallization. I have advised against desiccation of tooth structure by hot air, alcohol, or any such means preparatory to applying cement, and I have advised the *merest moistening* of a surface with *cement liquid* to facilitate the flow of the cement to intimate contact with all inequalities. I have also advocated treating with water and then drying reasonably any cavity surface suspected of being abnormally dry from protection by the rubber dam for an adjoining operation, and the treatment by sodium carbonate solution followed by distilled water and absorbent cotton of any surface possibly contaminated by mucus or medicines.

A cement, such as Ames' C. & B. or Inlay, when mixed to a *putty like consistency for filling purposes*, may best be treated to moisture immediately after packing and approximately shaping the plastic mass and having the patient bite upon tin foil to save trimming for occlusion, the final shaping and finishing being completed after the cement has become decidedly crisp under moisture.

The Ames' powders compounded with "C" liquid give prompt setting, no perceptible porosity in eosin solution no perceptible shrinkage or expansion in glass tube tests, and a very slight expansion as measured in a Black micrometer. The adhesion to tooth structure and metal and porcelain is such that much use has been made of the combination in the attachment of inlays, crowns, bridges and orthodontia appliances.

During my early clinical use of these quick-setting hydraulic cements a need was recognized, mostly in midsummer, of a slower setting combination. It was my custom to use and recommend the Harvard or Justi liquid with the Ames powders under such conditions. Later Ames D. & E. liquids were prepared to meet such needs.

The Ames' powders, as used with the Ames' "D" liquid, give masses showing a very slight porosity, the merest perceptible expansion as measured by the micrometer and provide a grade of adhesiveness sufficiently above the quicker setting combinations to warrant the expenditure of the extra time its use requires in the attachments of some precarious appliances

The special advantage accruing from the use of such a liquid is from. expansion being practically nil, which suggests its use in attaching porcelain inlays, with which less mechanical retention is offered than in cases of metallic inlays, crowns, and orthodontia appliances. With any of the latter a slight expansion decidedly enhances the result, while practically all porcelain restorations built to the most approved cavity preparations, need a cement which will set without shrinkage "as a matter of course" and one with which the expansion is practically nil.

Ames' "C" and "D" liquids may be blended in any proportions called for by setting desired, deriving in such use extra adhesion in proportion to the percentages to the blend. A practical means of rendering Ames'

oxyphosphate of copper slower of setting is by blending Ames' "C" liquid with that of this cement to the extent needed for proper retarding in midsummer.

The Ames' "E" liquid with the same powders will give a mass with very slight evidence of porosity, no shrinkage or expansion in either the wet or dry state, as there is internal compensation, because of which there is no peripheral change. A cement resulting from the mixing of this liquid with a proper heavy zinc oxide will set slowly at ordinary or subnormal temperature. Under tropical, or other similar conditions, this combination will set rapidly after crystallization begins. At any season or under any reasonable conditions, the setting of such a cement will be accelerated by the normal heat of the human tissues, and the setting can be further accelerated by an elevation of temperature from any consistent source.

Ames' "A" liquid will give so rapid a grade of setting that its use is not advised, in this connection, except for fillings needing rapid manipulation. It will provide an ultrahydraulic quality, thereby rendering possible some operations which would be impracticable with a slower setting cement or a cement seriously damaged by saliva during its setting.

These "E" and "A" liquids may be blended in equal parts, or one "E" and two "A" to give a medium setting liquid superior to any other known to me, unless the operator would go so far as to incorporate with this a proportion of Harvard, Justi, or Ames' "D" liquid, as, for instance, two parts "E" and one part "A" and one, two or three parts "D," depending on the degree of retarded setting desired. An acid solution containing the modifying ingredients of "E" and "A" liquid, however, would be of no value if put out as a single liquid, because of precipitation taking place in a few days which precludes the use of this mixture except as blended by the operator.

The Fellowship and Caulk cements, each of less than my prescribed age, were included by Dr. C. M. McCauley in a recent paper in the list of four "most called for at the depots," the other two being the Harvard and Ames, the Justi not happening to be included in this classification. Of these cements I had little clinical knowledge previous to my retirement from regular practice, because of experimental tests which did not warrant their clinical use. Shrinkage and brittleness, put these cements with me beyond extensive clinical consideration and restrict my comments.

I do not consider that the question of the reliability of oxyphosphate of zinc cements for the purposes for which they are used, can be summed up or dismissed by basing conclusions on what we may think of any one manufacture, as in that case we might say that such cement has been reliable from soon after the early beginning of experimentation with and use of it, on the ground that there has been the Justi and some closely allied products showing a sufficiently good clinical record from almost the beginning of oxyphosphate of zinc cement history. We might say from other observations that crown and inlay operations are wholly unwarranted.

The average result of cement operations as they are "observed" is the basis of opinion, rather than the "possible" result, which the observer often knows not of. It is, therefore, not surprising to often note references to the unreliability of oxyphosphate of zinc at the cervical margin when used as a filling material, and to its being the weak "link in the chain" when used in attachment of crowns and more especially of inlays.

It is the misfortune of the dentist and his patients that for various reasons the few safe cements have not been used as exclusively as their merits would warrant, as is indicated by the Justi preparation having been omitted by Dr. McCauley from his list of cements submitted to tests.

The "Items of Interest," Novemeber, 1910, printed from Dr. Fred. S. Bell, a paper, "Practical Hints on C & B Work," and "The Cosmos," May, 1911, felt justified in quoting a paragraph in its Periscope, which reads:

"A tooth which is to be crowned should never be filled with cement when the carious portion lies anywhere near the gum line, because that cement is sure to become disintegrated by the fluids of the mouth, and some day the crown will either come off because of the disintegrated cement or the tooth will need to be removed entirely because of recurring caries under the crown. In such cases amalgam should be used."

It is my understanding that orthodoxy in crown construction calls for carrying the band of a crown to and slightly beyond any cavity margins but even without this precaution, I know of several brands of cement with which such fillings might be exposed with safety.

In the discussion of Dr. Conzett's paper—"The Gold Inlay" N. D. A. in "The Cosmos," December, 1910, page 1369—Dr. Gallie says: "In my opinion we cannot place full confidence in this operation until in some way we have overcome the weakness of the cement, and have some one produce a cement that can be relied upon." Dr. Conzett says in another paper on the same subject when shrinkage of the gold inlay is considered (Items of Interest, July, 1911, p. 487). "It is true that the cements now given us by the manufacturers are of so good a quality that they will preserve a tooth for a long time, even though there is a slight discrepancy between inlay and filling, but it is not a safe proposition to stake one's reputation upon; therefore I prefer to take the safe side and form the inlay with gold in as small a mass as possible consistent with the best results. This only applies to very large masses, as I have said, for in the ordinary inlay the contraction is negligible." Although a bit foreign to the subject I cannot refrain from saying that if the large and complicated (sometimes compound) inlays were cast in sections and soldered together, this contraction, and resulting misfit would be obviated.

The advisability of some operations does hinge on the dependability of cement, and I feel that the advisability of cementation of a large class of fixtures was settled more than a decade since by some of the pioneers in the making of metallic inlays by the burnished matrix, thus paving the way for the Taggart, Barnes and Alexander methods.

I feel quite sufficiently sure that there has been a marked improvement in the cements of this class year by year since the beginning of their use, and that were crown and inlay operations commendable in their construction in proportion to the possibility of safe cementation, and were the safe cementation assured by intelligent selection and use of available cements, the patient would be very satisfactorily served.

Oxyphosphate of zinc cements cannot be made proof against mismanagement, and it can be said that a quick-setting cement is in more danger from mismanagement than one of slow-setting tendencies. For this reason it is to be regretted, to an extent, that the satisfactory behavior, under favorable conditions of certain quick-setting cements has so impressed many operators that it is difficult to successfully argue the need of eclectic use of quick and slow setting products. In an eclectic use, the operator should be possessed not only of a knowledge of the working properties of different proprietary products, but, if it happens that an operation calls for properties found in the liquid of one product, and not in the powder of that product, but in that of the product of another manufacturer, he will be much nearer a master of the situation if he has experimentally blended these different ingredients and is familiar with the results of all such blends. Pay no attention to the "Caution" often offered, "do not use any other liquid with this powder" or "do not use any other powder with this liquid," but consider that it is quite possible that advantages may accrue from such blends. There is no fatal incompatability between any cement liquid and the powder of any other cement of the same class. There is only to be considered whether the result of a blend is a desirable one.

A definite combination of a liquid of a certain composition and specific gravity, with a powder of a certain composition and fineness of comminution may be ideal for a certain class of operations under certain conditions, but far from ideal for other classes of operations.

The liquids and powders of different cements may be classified to advantage in groups according to similarities. Justi, Eisfelder, Harvard and Ames "D" liquids are so similar that they might be used interchangeably. Ames' "C" and Caulk's more recent liquids are similar. Ames' "A" and Fellowship liquid are similar. The first group give slow the second medium, and third, quick-setting, if mixed with a given zinc oxide powder. Silex Email liquid and Ames' "E" are similar. Classification of cement powders would place Justi's, Eisfelder's and Silex Email together as not very finely ground heavy zinc oxide. Harvard powder having the heavy zinc oxide considerably modified by silicious material, and having in addition a phosphatic modification places it in a class of its own. Ames' Crown and Bridge and Caulk's Improved Petroid and Caulk's Crown and Bridge powders are somewhat similar in pigmentation and fineness of division. I cannot class the Fellowship powders with any other here mentioned.

In the use of the word similar, I do not mean to convey that these similarities are in all cases definite. Two powders, for instance, might be so similar in composition, fineness of division, and in pigmentation that little difference would be noticeable in the mix, and yet one would result in a mass of satisfactory density and toughness, and the other a mass more or less dense, but very brittle. One might be safe at a cervical margin and the other hazardous. In this connection it is well to mention that a brittle cement is apt to show shrinkage during setting.

I am accustomed to seeing tests of breaking stress of cements appearing quite similar in the mix, in which one breaks at about 100 lbs. into a large number of small particles, while another stands 150 lbs., and in breaking gives only three or four parts, showing greater integrity with less brittleness.

. This classification of similarities ought to be of assistance to any operator wishing to observe the results of making mixtures of the powders and liquids of various products. For instance, the Harvard cement entire, or that powder with Justi, Eisfelder's or Ames' "D" liquid gives plasticity which might be considered very satisfactory, when mixed stiff for filling. These mixtures for filling purposes might be considered almost ideal were a percentage of such a powder as the Ames' Crown and Bridge blended into the mixture to the extent of 25% to add a sufficiently impalpable binder, the coarse powder needing such a blend. It is my impression that Harvard powder produced today is not as fine as it was at the time of its introduction. For giving a placticity suited to crown or inlay setting, the Harvard, Justi, Eisfelders and Silex Email cements, seems to me to be far from ideal, because of the comparatively coarse condition of the powders. The Justi, Eisfelder, Harvard and Ames "D" liquid, mixed with such a powder as the Ames Crown and Bridge, will result in a mass of very smooth plasticity, moderately slow setting tendencies, and decided adhesiveness without an observable degree of expansion during setting. Following out this blending in the direction of quick-setting combinations through that obtained by mixing a finely ground powder with such a liquid as the Ames "C" or the Silex Email for moderately quick setting in crown setting operations favorable to quick setting, and farther to the use of liquids giving still quicker setting warranted and called for in operations which can only be successful with such quick setting, you will have seen results which must perforce be of service to you in using such cements as demanded by the case in hand.

If I can by this effort convert a considerable number of operators to having at hand the requisite variety of cement powders and liquids for this eclectic practice, I will feel repaid.

I have said that a slow setting cement is less apt to suffer from mismanagement than one with quick setting tendencies. A cement with quick setting tendencies is mostly in danger of being mismanaged under such unfavorable conditions as extreme heat and humidity, therefore, a cement with *slow setting tendencies* must be relied on during the prevalence of the worst external governing conditions. In addition operators in countries subject to extreme summer heat find that a very early morning appointment is the most satisfactory means of minimizing the difficulties of a complicated cement operation in midsummer. Low temperatures are easily managed.

While our subject is oxyphosphate cements we have needed to occupy so much time over the zinc class that oxyphosphate of copper must be excluded from further mention.

I have no doubt that silicious oxyphosphates are uppermost in your minds, as the signs of the times point to the silicious cement question being one of almost paramount importance. Such a material is ideal if reliable.

You have been informed that these cements are oxyphosphates depending on calcium as the potent cement making ingredient. Formulas more or less explicitly and more or less correctly given in patent specifications make the ingredients of these compounds calcium and aluminum silicates with in one case a modification consisting of the silicate of a rare element.

Phenakit (German), Phenacite (English), is a natural beryllium silicate found in localities widely scattered The name Phenakit applied to a cement would naturally suggest a beryllium content. I do not know what is claimed by the makers of this silicious cement. I can only say that there is not enough of this element contained in any form to show in an analysis.

The name Berylite in connection with a silicious cement giving translucency as compared to a zinc preparation but not having sufficient translucency to become popular, was not intended to indicate a beryllium content. The name was adopted before the beryllium argument had been sprung, and was chosen as an euphonious term indicating jewel-like texture.

I happen to be in position to offer the opinion that when any maker advances a claim in regard to Beryllium or any other rare element being a controlling factor making for superiority and supremacy, it is merely grand-stand play, and that the rare element simply replaces aluminum without advantage except from the advertising standpoint.

The manufacturers of the latest silicious cement aspiring to prominence lay most stress on synthetic preparation being the greatest assurance of a safe and useful material, because of greater assurance of purity of ingredients. Given a commendable formula and manufacturing technic, purity of ingredients *will* save the manufacturer and user many annoyances and apologies. Iron, so prevalent in nature, is the bane of the producer of silicious cements. In the production of powders for oxyphosphate of zinc a considerable trace of iron, nickel, bismuth or lead may be contained in a compound oxide without danger of contributing dark colored sulphides, while any of these prevalent impurities as oxides or silicates as produced in nature's laboratory are fatal when contained in a dental silicious oxyphosphate because of the objectionable complexion of the sulphide of any of these metals and the inevitable formation of the sulphide under oral conditions. It is astonishing that one preparation, the Ascher, otherwise showing evidence of a carefully worked out formula and careful technic in compounding should yet show that this matter of freedom from the elements yielding these objectionable sulphides has not been secured. In this instance it is difficult to believe that compounds of these metals have not been used as pigments, as the plain white unpigmented material is practically free of discoloration in the mouth and all departures from this plain white show discoloration more or less seriously.

Admitting that silicious cements may be so compounded that no objectionable complexion will develope, the question then arises whether the frequent death of pulps beneath such cements is a necessary result of the use of the material or an unnecessary accident preventable by proper operative procedure. The natural early belief was that this pulp poisoning was from an arsenical ingredient. It is not reasonable to suppose that arsenic in any form would be contained in a material of this nature, and analyses do not show any such contamination. The most reasonable suspicion is that conditions during setting are favorable to pulp irritation from the phosphoric acid.

Translucency of these cements is apt to depend on fusibility of the powder, and fusibility has been obtained by either a phosphate or fluride ingredient. A phosphatic ingredient in a cement powder is necessarily conducive to slow setting, and slow setting with its continued free acid condition is a menace to a vital pulp.

Quickened setting as obtained by elevated temperatures with any of these silicious cements cannot be considered detrimental, but on the contrary I believe it is beneficial from the standpoint of integrity of mass. A quickened setting will in all probability lessen the danger of pulp devitalization.

The paper of Dr. Max Kulka as translated for The Items of Interest, May, 1911, has much to say on electrolytic dissociation, hydrolysis and ionization. After many abstruse chemical arguments he shows the possibility of penetration of plates of ivory by phosphoric acid and that free phosphoric acid is in evidence in some mixed silicious cements for a longer period than in some others, making the deduction that safety of the dental pulp depends on prompt and complete setting.

I happened to ascertain some years since that various fluo-silicates gave cement making properties with zinc oxid and astonishingly rapid setting with silicious cement powders. These fluo-silicates used in conjunction with a heavy acid phosphate solution proved valuable as the liquid element of the material called Berylite, it being possible by varying the proportion of the two solutions to obtain any setting quality desired. While for various reasons I have not been hasty in seriously offering a silicious cement to the profession, I have often suggested the use of this mixture of phosphate and fluo-silicate to be used with powders obtainable. The combination gives improved texture and more definite setting and therefore greater safety for the tooth pulp along with expedition of the operation. Schoenbeck, Ordel and Phenakit powders may be used in this way.

Prompt setting may be obtained from a very potent powder and a not very energetic liquid or by the reverse of this, or by having the powder and liquid each moderately energetic in cement forming tendencies. In my opinion the latter condition represents the well balanced formula if the setting tendencies allow of mixing to a full puttylike consistency.

The Ascher cement when the use of some of its shades is justified in a clean mouth will set promptly to a dense mass when extra heat is applied, because of powder and liquid each being moderately active. Harvardid will do the same from having an energetic liquid with a rather inert powder. De Trey's will set promptly from a comparatively plastic mass and too quickly, to a porous mass, after a putty-like mix because of a very active liquid and a very inert powder. Ordell's is a bit slow because of a rather inactive liquid with a powder capable of medium activity. Schoenbeck's is a bit slower than Ordell's for similar reasons Phenakit is very slow, having a powder capable of moderate activity and a liquid not at all adapted in my opinion to the purpose.

These descriptions of my findings will, I trust, be of some assistance to you.

I have seen results which would indicate that silicious cements obtainable can be depended on for practically permanent results in the classes of cavities in which we most need them, and that in classes of cavities in which we would not expect real permanency because of the stress of mastication, they may be depended upon for a much longer period than any oxyphosphate of zinc.—*Journal of Allied Societies*, December, 1911.

